# High HIV Burden in Men Who Have Sex with Men across Colombia’s Largest Cities: Findings from an Integrated Biological and Behavioral Surveillance Study

**DOI:** 10.1371/journal.pone.0131040

**Published:** 2015-08-07

**Authors:** Martha Lucía Rubio Mendoza, Jerry Owen Jacobson, Sonia Morales-Miranda, Clara Ángela Sierra Alarcón, Ricardo Luque Núñez

**Affiliations:** 1 United Nations Population Fund, UNFPA, Bogota, Colombia; 2 Pan American Health Organization, Bogota, Colombia; 3 Centre for Health Studies, Del Valle University, Guatemala City, Guatemala; 4 Ministry of Health and Social Protection, Bogota, Colombia; The University of New South Wales, AUSTRALIA

## Abstract

**Background:**

Among Latin America’s concentrated HIV epidemics, little is known about men who have sex with men (MSM) in Colombia, the region’s third largest country. To date, surveillance studies have been limited to Bogota, while 80% of HIV cases and deaths originate from Colombia’s other cities and departments. The extent to which interventions should prioritize MSM outside of Bogota is unknown.

**Methods:**

We recruited 2603 MSM using respondent-driven sampling from seven of Colombia’s largest cities. HIV prevalence was estimated by site from dried blood spot samples. Behavioral data were collected through face-to-face interviews and risk factors for HIV infection analyzed using weighted, multi-level logistical regression models accounting for recruitment patterns.

**Results:**

Across cities, HIV prevalence averaged 15%, varied from 6% to 24% and was highest in Cali, Bogota, and Barranquilla. In the past 12 months, 65% of MSM had ≥ 5 casual male partners and 23% had a female partner. Across partnerships (i.e., casual, stable, and commercial), the proportion of MSM engaging in unprotected sex was ≥ 52% with male partners and ≥ 66% with female partners. Self-reported history of STI (24%) and past-year illicit drug use (38%) were also common. In multivariate analysis, age ≥ 35 (adjusted odds ratio [AOR], 19.2) and 25–39 (AOR, 5.6) relative to ≤ 18–24 years, identifying as homosexual relative to heterosexual (AOR 0.1), meeting casual partners on the Internet (adjusted odds ratio [AOR], 3.1) and age of sexual debut of ≤ 13 years (AOR, 3.1) predicted HIV infection. HIV testing and prevention messaging reached just 24% of MSM in the past year.

**Conclusions:**

Findings support consistently elevated HIV burden among MSM throughout Colombia’s largest cities and a need for enhanced behavioral prevention and HIV testing, emphasizing men who use the Internet as well as physical venues to meet sex partners.

## Introduction

Colombia is the second most populous nation in South America after Brazil and the third largest in the Latin America region, with 48 million inhabitants [1]. The country’s estimated 150,000 people living with HIV (PLWH) account for 10% of all PLWH in the region [2]. Yet, while in several Latin American nations men who have sex with men (MSM) have been identified as the population most central to HIV transmission [3,4,5], regular surveillance studies in Colombia have centered on women attending antenatal clinics, which have consistently demonstrated HIV prevalence below 0.5% since 1989 [6].

Available data on HIV among MSM date from a single study in 2002 in Bogota, the national capital, which reported a prevalence of 20% (N = 660) [7]. This was among the highest of estimates from a regional surveillance initiative in 36 South American cities. Since 2002, the annual number of reported HIV cases has increased nationally, from approximately 3–5 thousand cases through 2007 to 6–7 thousand cases from 2008 to 2010 [6]. HIV mortality has also increased, in contrast to trends elsewhere in the region [8], despite a national policy providing for freely available antiretroviral therapy since 1999. Amidst this expanding epidemic, male HIV mortality has consistently outpaced female mortality (8.1 vs. 2.1 per 100,000 people in 2011) and 72% of HIV cases are reported among males [6], suggesting the potential importance of MSM in HIV transmission. However, the recent trajectory of HIV among MSM and the situation beyond Bogota, which has historically accounted for just 20% of reported HIV cases and AIDS deaths [9,10], is unknown.

In Colombia, HIV prevention activities targeting MSM are planned and implemented by local public health departments and non-governmental organizations (NGOs), rather than being coordinated through a national agency or plan. In general, prevention among MSM consists of venue-based outreach to provide information on safe sex practices, motivate individuals to undergo HIV testing, and distribute free condoms. HIV testing services are available through a range of public and private health facilities, however there are no specialized clinics or testing services specifically for MSM. Fees for HIV testing are discounted or waived for individuals enrolled in one of the national healthcare system’s (*Sistema General de Seguridad Social en Salud* [SGGSS]) insurance plans, based on non-employment or income thresholds. Access to the SGGSS involves an enrollment process with an authorized public or private insurance agency. Research has identified enrollment and higher educational attainment as correlates of HIV testing in the general population [11].

**Fig 1:**
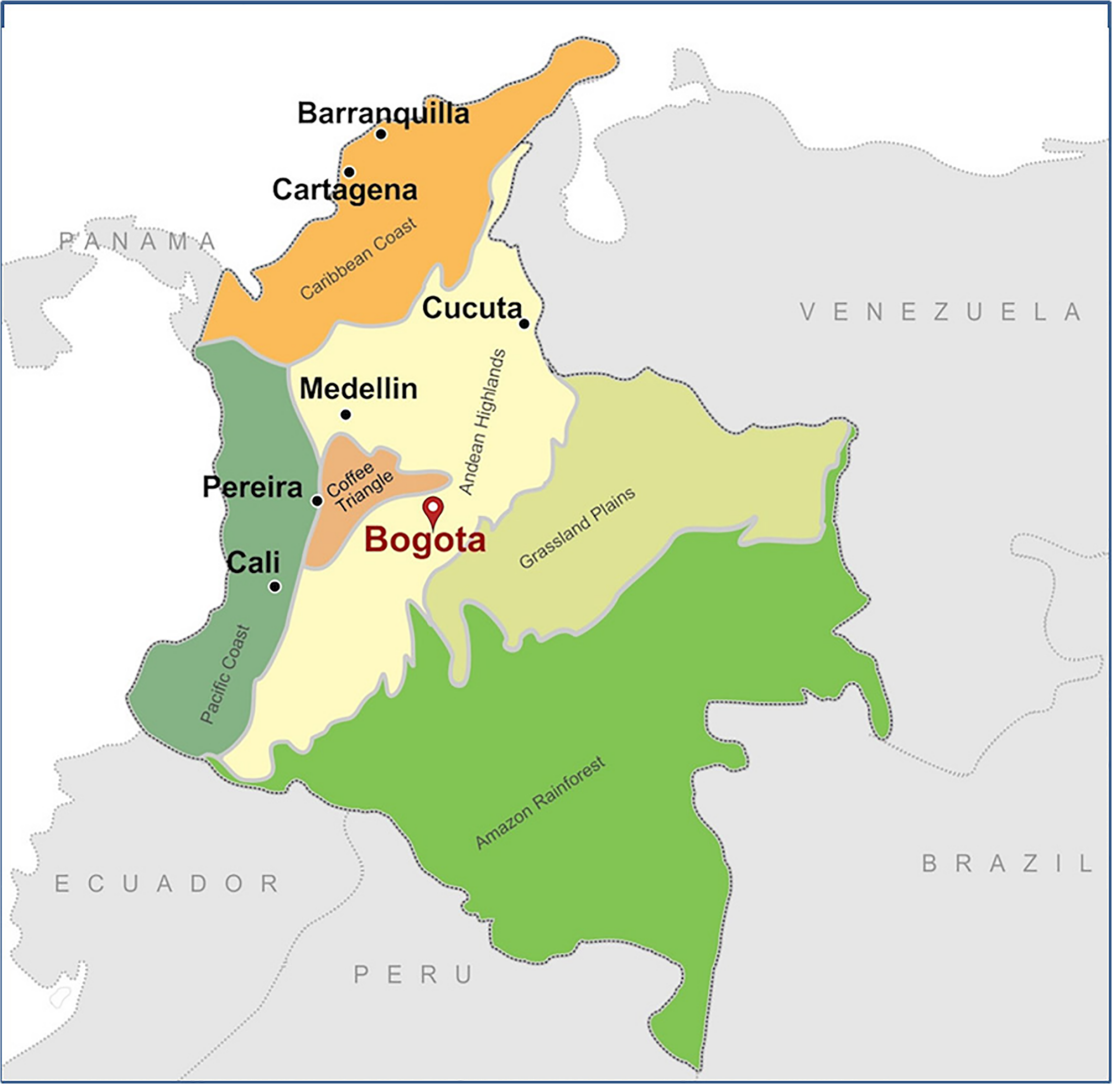
Cities included in the study. Shading indicates the geographic regions in which the study cities are located.

This paper presents findings from an HIV and behavioral surveillance study among MSM in seven of Colombia’s largest cities, which together represent 33% of the population and include the Andean Highlands, Pacific and Caribbean coastal regions (Fig 1) [9]. The paper aims to characterize levels of HIV infection across cities and, in the joint sample, to assess the prevalence of sexual and drug risk behaviors and the coverage of testing and prevention. Finally, we identify factors associated with HIV infection in order to inform the targeting of prevention efforts.

## Materials and Methods

### Ethics Statement

The study protocol was approved by a *ad hoc* ethics committee of Colombian public health and HIV professionals convened by the United Nations Population Fund in Bogota. Written consent was obtained from all participants prior to study enrollment. Names collected to link participants with a positive HIV diagnosis to care through the national health system and as part of the consent process were permanently de-linked prior to data analysis.

### Recruitment

Study participants were recruited by respondent-driven sampling (RDS), a chain referral method designed for hard-to-reach and stigmatized populations [12,13], which has been utilized widely for HIV surveillance in Latin America [14] and globally [15]. Formative research ahead of the main study consisted of qualitative interviews with 15–20 MSM by local community organizations to inform the choice of study site location, hours, personnel, incentive levels, and to identify 3–7 potential “seeds” who would initiate recruitment in each city, selected to achieve diversity with respect to age, education and previous HIV diagnosis.

Men who were 18 years of age and older, who reported having oral or anal sex with another man in the past 12 months, resided in the respective study city and who were in possession of a recruitment coupon were eligible to participate. Individuals who identified as female, transgender or transsexual, were not eligible given plans for a separate surveillance study in this population.

Following completion of interview and collection of blood sample, participants were provided with three recruitment coupons to be offered to peers whom they believed likely to satisfy eligibility criteria. Coupons listed information about the study site in each city—a centrally located, unmarked apartment or house—and a code to track recruitment. Subjects received information on sexual health and the equivalent of US$10.50 for their participation and could receive a one-time secondary incentive of condoms and lubricants if at least two referrals completed the survey interview.

We planned for a sample size of 504 MSM in Bogota and 350 MSM at other study sites (Table A in S1 File).

### Data collection

Participants completed a face-to-face survey interview adapted from standard HIV behavioral surveillance instruments [16]. The questionnaire covered a range of themes including social and demographic characteristics, sexual relationships, consumption of alcohol and illicit drugs, use of condoms and lubricants, knowledge, testing and treatment-seeking related to STIs and HIV, and experiences of stigma related to sexual orientation. Respondents were asked about male and female partners. “Stable” partnerships were defined as having lasted at least six months and “casual” partnerships less than six months, an arbitrary threshold to ensure a common definition across respondents; “stable” and “casual” partners were further defined as not involving cash payment in exchange for sex, in contrast to “commercial” partners. The time frame for most questions was the past 12 months, while questions on drinking and on condom use with female partners, casual male partners and sex clients referred to the past 30 days.

Following HIV and STI counseling, finger-prick dried blood spot samples were collected and processed at a centralized laboratory. Assessment of HIV status followed the national algorithm: an initially positive enzyme immunoassay (ELISA) was followed by a second ELISA, which, if positive, was confirmed by Western Blot. Participants were asked to return following 10 days for test results and post-test counseling. Following completion of the study, results were made available through the respective city’s Secretary of Health office, which conducted follow-up by phone to participants who had not retrieved their results and had voluntarily provided a contact phone number.

### Study Measures

The National Statistics Department’s neighborhood-level measure of socio-economic status, *estrato socioeconómico*, was used as a proxy for the participant’s socio-economic status [17]. The *estrato* is assessed by the city-level government on a six-point scale (1 [“low-low”], 2 [“low”], 3 [“middle-low”], 4 [“middle”], 5 [“middle-high”] and 6 [“high”]), based on the physical characteristics of a neighborhood’s housing infrastructure. In Colombia, individuals are generally aware of their residential neighborhood’s *estrato* because the cost of municipal public services such as electricity, gas and water are a function of it. However, there is no published equivalent to individual or household income.

Participants were asked to describe their cultural identity as one of a series of pre-defined response categories. Responses of “Indigenous” and “native to the San Andres and Providencia islands” were classified as Indigenous; responses of “Black, Mulato, Afro-Colombian or Afro-descendent” and “*Palanquero de San Basilio”* were classified as Afro-Colombian; and responses of “Romani” and “none of the above” were classified as Mestizo/other.

A measure of internal displacement by violence was defined as reporting having relocated to the present city of residence primarily because of a threat on the participant’s life, freedom or physical health due to the country’s on-going internal armed conflict. Forced internal displacement is a well-known phenomenon in Colombia and estimates of the number of displaced range from 3.7 million people from 1997 to 2011 to 5.3 million from 1985 to 2011, and from 100,000 to 300,000 people in year 2010 alone [18]. Qualitative research suggests that forced displacement in Colombia may lead to vulnerability to sexual abuse and high-risk sexual relationships among MSM [19].

We assessed enrollment in the national health system as reporting generally accessing health care services by using any one of the health insurance membership cards in current use, including cards for the subsidized plan, the more expensive contributory plans, a reduced services plan designed for individuals unable to meet standards for the subsidized plan or pay for the contributory plans (*vinculados*), indigenous protection plan, and the police and military plan.

Sexual identity was self-reported as homosexual, heterosexual or bisexual. A measure of having been coerced into sex was defined as reporting having been forced to have sex with any sexual partner in the past 12 months. History of STI was defined as reporting having ever been diagnosed by a medical doctor with a sexually transmitted infection, including gonorrhea, chlamydia, syphilis, herpes, hepatitis B, condyloma or genital warts, or any STI without a specific diagnosis, excluding HIV/AIDS. As a measure of comprehensive HIV knowledge, we used the UNAIDS definition of correct responses to all in a series of five questions on HIV transmission and prevention [20]. The mean number of correct responses to the five questions was also estimated. Consistent condom use was defined as reporting “always” (as opposed to “most of the time”, “sometimes” or “never”) using a condom during intercourse with the respective type of partner.

A measure of personal network size (“degree”)—the number of other MSM who could have conceivably recruited the participant into the study [12,13]^—^was assessed through a series of questionnaire items as the number of other MSM with whom the participant had been in contact over the past six months, who were thought to be eligible for the study and likely to recruit the participant had they been provided a recruitment coupon. To correct inconsistent values, we fixed degree at the greater of (1) self-reported degree and (2) the number of referrals to the study, plus one representing the participant’s recruiter, a correction applied to 6.5% of participants. One missing degree value was imputed at the mean.

Three of the measures presented were characterized by relatively high levels of non-response, including daily consumption of alcohol (5.2% non-response), ever having been diagnosed with an STI (4.6%) and having received information on HIV in the past 12 months (2.6%).

### Statistical analysis

We developed HIV prevalence estimates by city using the RDS-1 estimator, which attempts to reduce selection bias due to recruitment patterns along observed characteristics and adjust for differential probability of selection as evidenced by differences in degree [12,13]. Confidence intervals were derived by the bootstrap method [21].

Our strategy for developing estimates from the sample pooled across cities builds on an approach recently introduced for similar multi-site RDS studies [19,20,22]. HIV prevalence and population characteristics were estimated as weighted proportions with exact binomial confidence intervals. Weights were inversely proportional to the participant’s degree, proportional to the urban male population ages ≥ 18 years in each city in 2010 (assuming this in turn is proportional to the MSM population), and were scaled to sum to the overall sample size.

We also constructed model predicting HIV infection using bivariate and multivariate logistic regression models. Models included statistical adjustments to correct for potential violations in independence assumptions. Specifically, we sought to identify recruiter-level variables associated with HIV infection at the 10% level, including recruiter’s infection status, demographics and sexual behaviors, while controlling for the participant’s value of the same. Recruiter’s HIV infection proved significant (*P* = 0.092) and was therefore retained in the bivariate and multivariate models, together with a recruiter-level random effect, which improved the fit of the final multivariate model according to likelihood ratio tests [23].

The multivariate model was initially estimated by including variables with a significant bivariate association (*P* ≤ 10%) with the outcome and study site fixed effects. Interactions between main effects were evaluated. Variance inflation factors from linear specifications of the model were < 2, indicating the absence of multicolinearity problems. Risk analysis was limited to variables on which ≥ 25 participants provided an affirmative response. We considered strong associations as *P≤ 5%* and marginal associations as *P ≤ 10%*, both of which were retained in the final model. All analyses excluded seed participants and were conducted in Stata 12.0 (College Station, TX).

Findings from a preliminary analysis of these data appear in a Spanish language national study report, which presented univariate estimates of population characteristics by city and estimates stratified by HIV infection status for the pooled sample [24].

## Results

### Recruitment

From May to November of 2010, 36 seeds (3–9 seeds per study site) recruited a total of 2567 non-seed MSM participants (ranging from 333 to 488 per study location) (Table A in S1 File). Of the 36 seeds, 6 tested positive for HIV infection, 16 were age ≥ 35 years, 11 were ages 25 to 34 and 9 were ages 18 to 24 (Table B in S1 File). Nearly all seeds had completed secondary education (N = 18) or higher (N = 17). Data collection lasted from 1.5 months in Pereira to 4.3 months in Bogota. The longest recruitment chains reached between 11 and 16 waves in each city and equilibrium was attained on all variables examined [25].

### HIV burden

Specimens for HIV testing were provided by 99.6% of participants (from 99.4% to 99.8% across sites). The overall estimated proportion of MSM infected with HIV was 15.1% and ranged from 5.8% in Cucuta to 23.7% in Cali (Table 1). Confidence intervals suggest a high likelihood that HIV burden in Cali and Bogota, and to a lesser extent Barranquilla, are higher than in the other cities. Given the similarity of the crude and RDS-adjusted estimates, which differed by at most three percentage points, the remaining analysis focuses on the adjusted estimates.

**Table 1:** Prevalence of HIV infection among men who have sex with men in Colombia by study site.

Study site	n/N	Crude percentage	RDS-adjusted percentage	95% CI
Cali	76/331	23.0	23.7	17.6–29.9
Bogota	79/487	16.2	16.3	11.4–21.7
Barranquilla	49/349	14.4	15.3	9.4–21.8
Cartagena	32/349	9.2	8.5	5.1–12.4
Medellin	39/348	11.2	8.2	4.7–12.4
Cucuta	28/348	8.0	8.0	4.2–12.9
Pereira	27/346	7.8	5.8	2.7–9.5
All study sites	330/2558	^^^15.4	^§^15.1	12.6–17.6

^^^ Pooled estimate, weighted by male population aged ≥ 18 years in city.

^§^ Weighted by population in city and inverse degree. CI, confidence interval.

### Socio-demographic characteristics

About half of MSM in the cities examined were younger than 24 years old (48.3%) and 76.2% were younger than 35 years (Table 2). While most had completed secondary education (77.9%) and were employed (61.9%), 49.0% resided in neighborhoods belonging to the poorest socio-economic strata. The vast majority identified ethnically as *mestizo* (89.0%) and 9.1% as Afro-Colombian. Overall, 78.6% were enrolled in the SGGSS.

**Table 2:** Social and demographic characteristics of MSM in Colombia.

	n/N	RDS-adjusted percentage	95% CI
**Age**			
18–24	1356/2567	48.3	44.9–51.8
25–34	738/2567	27.9	24.9–30.9
≥ 35	473/2567	23.8	20.6–26.9
**Highest education attained**			
Primary or below	512/2567	22.1	19.0–25.1
Secondary	1470/2567	53.2	49.7–56.7
Vocational, university or above	585/2567	24.7	21.7–27.7
**Socio-economic stratum**			
Low-low to middle-low (0–2)	1335/2531	49.0	45.5–52.5
Middle-low (3)	878/2531	36.1	32.8–39.4
Middle to high (4–6)	318/2531	15.0	12.3–17.6
**Employed**	1581/2567	61.9	58.5–65.3
**Cultural identity**			
Mestizo / other	2275/2567	89.0	87.1–91.0
Afro-Colombian	254/2567	9.1	7.4–10.8
Indigenous	38/2567	1.9	0.9–2.9
**Internally displaced by violence**	38/2567	2.4	1.1–3.7
**Enrolled in the national health insurance system**	2072/2567	78.6	75.6–81.6

Notes: Number of respondents in the respective category (n) and total number of respondents (N) exclude seeds. Differences in denominators reflects non-response.

### Sexual identification and behaviors

The sample led to estimates of the MSM population as largely homosexual- (67.5%) and bisexual-identified (27.1%), with 5.4% identifying as heterosexual (Table 3). The median age of initial male-to-male sexual intercourse was 16 years (unweighted, not shown) and 20.6% began at 13 years or younger. About three in ten (30.6%) of MSM were in stable relationships with male partners and during the 12 months prior to the survey, 65.4% had had oral or anal sex with five or more men, 10.4% had sold sex to a male partner and 5.6% had been forced to have sex. The estimated percent of MSM who consistently used condoms during anal sex was 34.2% with stable male partners, 48.4% with casual male partners, and 68.0% with male sex clients.

**Table 3:** Sexual identity and relationships and alcohol and drug use among MSM in Colombia.

	N	RDS-adjusted percentage	95% CI
**Sexual identity**			
Homosexual	1740/2560	67.5	64.2–70.7
Bisexual	693/2560	27.1	24.1–30.2
Heterosexual	127/2560	5.4	3.8–7.0
**Age at first male-to-male sex (years)**			
≥ 18	582/2567	37.0	33.7–40.4
14–17	1139/2567	42.3	38.9–45.8
≤ 13	846/2567	20.6	18.0–23.2
**Sexual relationships**			
Has stable male partner	747/2560	30.6	27.4–33.8
Has stable female partner	154/2566	5.8	4.4–7.1
Any recent female partners^‡^ ^,^ ^^^	565/2567	22.7	19.8–25.6
Paid for anal sex^‡^	114/2567	3.8	2.4–5.1
Received money for anal sex^‡^	287/2567	10.4	8.4–12.5
Coerced into sex^‡^	154/2562	5.6	4.0–7.1
**No. recent male partners** ^‡^ ^**,**^ ^^^			
1	579/2567	26.1	23.1–29.1
2–4	258/2567	8.5	6.7–10.2
≥ 5	1730/2567	65.4	62.2–68.7
**Consistent condom use** ^§^			
Anal sex with stable male partner^‡^	218/687	34.2	27.7–40.6
Anal sex with casual male partners^||^	964/2049	48.4	44.5–52.3
Sex with stable female partner^||^	11/154	4.9	1.2–8.7
Sex with casual female partners^||^	181/465	34.0	26.6–41.3
Anal sex with male sex work clients^||^	187/284	68.0	58.6–77.4
**Met casual male sex partners via** ^‡^ ^**,**^ ^§^			
Bars, dance clubs, night clubs	726/2281	29.6	26.4–32.9
Public places (street, parks, transport)	546/2281	23.1	20.1–26.1
Internet websites	597/2281	22.3	19.6–25.1
Saunas / Turkish baths	131/2281	8.1	6.1–10.2
Adult theatres	126/2281	6.4	4.7–8.0
Internet facilities (*cabinas)*	115/2281	4.9	3.4–6.4
**Daily alcohol use** ^||^	22/2433	1.3	0.2–2.4
**Illicit drug use** ^‡^	905/2566	37.9	34.5–41.3

Notes: Number of respondents in the respective category (n) and total number of respondents (N) exclude seeds.

^||^ past 30 days

^‡^ past 12 months

^^^ includes commercial and non-commercial sex partners

^**§**^ of those who reported the respective type of sex partner. Other differences in denominators reflect non-response.

Casual male sex partners were met in diverse ways, with no single modality characterizing a majority of MSM. Bars and dance clubs were the most common meeting point (29.6%). Slightly fewer had recently met partners through the Internet or at public places.

Although just 5.8% of MSM were in stable relationship with a female partner, 22.7% had had female sex partners over the past year. Compared to male anal sex partners, consistent condom use with female partners was lower in both stable (34.2% vs. 4.9%) and casual (48.4% vs. 34.0%) partnerships. Past-year illicit substance use was common, with 29% of MSM consuming cannabis, 16% cocaine or crack and 13% ecstasy or “poppers”. Few had ever injected heroin (0.8%, 95% CI 0.0–1.8, not shown). A history of other STIs was common (24.3%).

### HIV knowledge and testing

Awareness of HIV/AIDS was nearly universal (Table 4) and on average MSM provided an estimated 3.9 (95% CI: 3.8–4.0) correct responses to the 5 UNAIDS standard knowledge questions, with an estimated standard deviation of 1.1 (weighted estimates, not shown in table). However, only 34.1% of MSM demonstrated comprehensive knowledge. About one quarter had utilized HIV testing (23.6%) or been exposed to HIV prevention (27.1%) in the past year.

**Table 4:** HIV and STI knowledge and testing among MSM in Colombia.

	N	RDS-adjusted percentage	95% CI
**HIV knowledge**			
Ever heard of HIV or AIDS	2499/2566	97.6	96.4–98.8
Comprehensive knowledge of HIV	920/2566	34.1	30.9–37.3
Received information on HIV in past 12 months	729/2499	27.1	24.0–30.2
**HIV/STI testing**			
Tested for HIV in past 12 months	614/2566	23.6	20.7–26.6
Ever diagnosed with an STI	545/2448	24.3	21.3–27.4

Notes: Number of respondents in the respective category (n) and total number of respondents (N) exclude seeds.

### Associations with HIV infection

Factors strongly associated with HIV infection in bivariate and multivariate analysis included older age (≥ 35 years [AOR 19.2] and 25–34 years [AOR 5.6] relative to 18–24 years), meeting casual sex partners on the Internet (AOR 3.1), age of male-to-male sexual debut of ≤ 13 years (AOR 3.1) relative to ≥ 18 years, and identifying as homosexual relative to heterosexual (AOR 0.1). Marginal associations were detected for MSM who met sex partners at saunas (AOR 3.0) or public places (AOR 1.3). Subgroups in which HIV infection was significantly more prevalent, but which did not persist in multivariate analysis, included MSM who were employed, had a stable male partner, had sold sex, were forced to engage in sex, met sex partners at adult theatres, had a prior STI diagnosis or consistently used condoms in recent relationships. Having a recent female partner was protective.

Adjusted HIV prevalence was considerably higher in Cali (AOR 2.9) and marginally higher in Barranquilla (AOR 2.2) and Bogota (AOR 2.0) relative to other cities, after accounting for differences in demographics and risk behaviors (Table 5).

**Table 5:** Associations with HIV infection among MSM in Colombia from bivariate and multivariate models.

	Prevalence of HIV infection ^§^	Bivariate models		Multivariate model(N = 2203)	
	% (95% CI)	OR (95% CI)	*P*	AOR (95% CI)	*P*
**Age**					
18–24	7.5 (5.3–10.4)	1.0	-	1.0	-
25–34	16.2 (12.6–20.7)	2.5 (1.8–3.5)	0.000	5.6 (2.4–13.1)	0.000
≥ 35	29.3 (22.5–37.3)	4.6 (3.2–6.5)	0.000	19.2 (7.2–50.8)	0.000
**Employed**	14.9 (12.2–18.1)	1.7 (1.3–2.3)	0.000		
**Internally displaced by violence**	39.0 (15.6–68.8)	2.2 (0.9–5.5)	0.077		
**Sexual identity**					
Homosexual	17.7 (14.6–21.2)	1.0	-	1.0	-
Bisexual	10.4 (6.7–15.8)	0.7 (0.5–0.9)	0.020	0.5 (0.2–1.1)	0.096
Heterosexual	6.6 (2.6–15.9)	0.4 (0.1–0.9)	0.024	0.1 (0.0–0.8)	0.033
**Age at first male-to-male sex (years)**					
≥ 18	10.9 (7.9–15.0)	1.0	-	1.0	-
14–17	13.8 (10.4–17.9)	1.2 (0.8–1.6)	0.374	2.1 (0.8–5.3)	0.122
≤ 13	25.4 (19.3–32.7)	1.8 (1.3–2.5)	0.001	3.1 (1.3–7.6)	0.011
**Sexual relationships**					
Has stable male partner	16.5 (12.3–21.7)	1.4 (1.1–1.9)	0.016		
Any recent female partners^‡^ ^,^ ^^^	8.5 (4.8–14.8)	0.5 (0.3–0.7)	0.000		
Paid for anal sex^‡^	20.1 (10.3–35.5)	1.6 (0.9–2.8)	0.080		
Coerced into sex^‡^	13.4 (8.1–21.3)	1.6 (1.0–2.6)	0.045		
**Consistent condom use**					
Anal sex with stable male partner^‡^	25.1 (15.8–37.5)	2.7 (1.5–4.7)	0.001		
Anal sex with casual male partners^||^	17.9 (14.2–22.2)	1.7 (1.3–2.3)	0.000		
Sex with stable female partner^|^	31.5 (9.2–67.6)	5.7 (0.8–41.7)	0.088		
**Met casual sex partners via** ^‡^					
Saunas	25.1 (16.3–36.5)	3.0 (1.8–4.9)	0.000	2.6 (1.0–7.0)	0.054
Adult theatres	25.0 (15.5–37.7)	2.3 (1.4–3.8)	0.001		
Internet websites	18.2 (13.3–24.2)	1.3 (1.0–1.8)	0.092	3.1 (1.2–7.6)	0.015
Public places (street, parks, transport)	19.5 (14.4–25.9)	1.3 (1.0–1.8)	0.071	1.9 (0.9–4.2)	0.095
**History of STI**	23.6 (18.1–30.2)	2.6 (1.9–3.4)	0.000		
**Study site fixed effects**					
Cali	-	-	-	2.9 (1.3–6.5)	0.012
Barranquilla	-	-	-	2.2 (0.9–5.2)	0.086
Bogota	-	-	-	2.0 (0.9–4.4)	0.103
Cartagena, Cucuta, Medellin and Pereira	-	-	-	1.0	-
**Recruiter infected with HIV**	-	-	-	3.8 (1.8–8.0)	0.000
**Variance of recruiter random effect (standard error)**	-	-	-	5.7(0.90)	-

Notes: Only measures with a bivariate relationship of *P ≤ 0*.*1* are shown. OR, odds ratio. AOR, adjusted odds ratio. CI, confidence interval.

^§^ RDS-adjusted estimates

^||^ past 30 days

^‡^ past 12 months

^^^ includes commercial and non-commercial sex partners.

## Discussion

In one of Latin America’s largest countries, this study provides the first characterization of HIV burden among MSM and subgroups at increased risk of infection in several of Colombia’s largest cities. Our main finding is that of consistently high levels of HIV, ranging from about 6% to 24% across cities. The overall prevalence, 15%, is identical to the regional average and consistent with the 14–18% range for MSM among middle- and low-income countries [3], thus adding to the evidence of the severe impact of the HIV epidemic on MSM in this region and globally. Particularly elevated levels of infection in Bogota, Cali and Barranquilla, which span Colombia’s Andean, Pacific and Caribbean regions, respectively, suggest that HIV among MSM must be considered a national problem. This stands in contrast to reduced prevalence levels among injection drug users (approximately 2%) and the general population at 0.5% [26,27].

Early diagnosis of HIV infection is critical to ensure timely linkage to care and treatment and to limit onward transmission. Yet, we estimate that only 23.6% of sexually active MSM receive HIV testing annually, despite much higher levels of unprotected sexual intercourse. Rates of recent testing in Colombia therefore stand at about half of the median across Latin American countries [28] and a third of levels in United States cities such as New York and Los Angeles [29,30], suggesting considerable room for expansion of testing services. This study underscores the need for operational research to reduce barriers to testing specifically among MSM as well as more detailed, city-level analysis of data from the present study. A recent mixed methods study identified a number of individual and structural factors related to HIV testing in Bogota [31]. In addition, introducing HIV rapid tests—in the context of health services and future surveillance studies—could make testing more attractive and reduce loss-to-follow up.

HIV prevention targeting MSM in Colombia generally consists of outreach to venues identified with the gay community. As our findings suggest elevated HIV prevalence among MSM who are gay-identified or meet casual sex partners at saunas, adult theatres, on the street or in other public places, geographic targeting of venues appears warranted. However, MSM who had recently met sex partners on the Internet presented a threefold increase in the log-odds of HIV infection, suggesting an elevated risk of transmission. Colombia experienced a dramatic increase in Internet users over the decade leading up to this study, from 2.5 to 36.5 per 100 people [32]. Anecdotally, social networking websites and geosocial networking phone applications similar to GRINDR in the US [29,30,33,34] are increasingly used by MSM. Therefore, it is critical that prevention strategies keep pace by identifying and targeting such sites to provide HIV and STI awareness messages, promotion of safer sex behaviors, and linkage to testing services, as has been undertaken elsewhere [29].

Nearly a quarter of this population had previously been diagnosed with STI’s other than HIV, a figure which does not capture undiagnosed infections. STIs elevate the sexual transmission risk for HIV infection [35,36]. Across the region, studies that have tested for STIs among MSM have consistently reported elevated prevalence [37,38,39].

Behavioral findings from this study suggest that transmission among MSM may be ongoing. Relatively few MSM were in stable relationships and a greater proportion of MSM reported numerous recent casual male partners, inconsistent condom use across types of partnerships, and recent illicit drug use relative to reports from elsewhere in the region [37,38,39,40]. The high proportion of MSM who had initiated same-sex intercourse at a young age may indicate an extended exposure period. While relatively few heterosexual-identified men were recruited into our sample, a high proportion of MSM had recently engaged in unprotected sex with female partners, suggesting the potential for transmission to the general population. As the study was cross-sectional, however, causal links between these behaviors and HIV infection cannot be established.

Our estimated HIV prevalence for Bogota of 16.3% (95% CI 11.4–21.7) in 2010 is not significantly different from an estimate of 12.1% (95% CI 8.7–15.8) reported by a 2011 study that also used RDS [41] and is similar to an estimate of 19.4% reported by a 2002 study [7]. However, assessment of trends is hindered by differences with the earlier study’s venue-based sampling strategy. As we did not assess HIV incidence directly, we can only infer the direction of the epidemic from the behavioral findings. To some extent, these behaviors may have been under-estimated, as our face-to-face survey administration design may have introduced social desirability bias [42]. Relatively high levels of non-response on questions regarding the frequency of drinking, history of STI, and having recently received information about HIV may have been due to such bias or, alternatively, to problems understanding the survey questions, in which case the direction of the bias is unclear. The low level of injection drug use may have resulted from asking exclusively about heroin injection, whereas cocaine is often injected in the region [43,44]. A further limitation is the study’s geographic coverage as locations were not selected randomly and the Plains, Amazon Basin and Chocó regions were not examined; differences in proximity to international borders and socio-demographic context could give rise to epidemiological differences in these areas.

The RDS sampling method succeeded in recruiting a large number of MSM at all locations in a time frame within the range recommended for HIV surveillance [45], and, by tracking linkages, allowed for corrections for selection bias. This study also contributes to statistical methods for pooled analysis of RDS data collected from multiple locations by demonstrating the need to consider study site fixed effects, which proved significant. Nonetheless, RDS estimation techniques are a subject of ongoing debate, are known to under-estimate variance (and as a result, test statistics) and do not always offer improvements over unadjusted estimates [46]. To the extent that the RDS estimation algorithm failed to correct for non-random selection, estimates could be biased. For example, the estimated 48.3% of MSM ages 18 to 24 would seem to indicate that youth were more likely to be sampled, which could lead to an under-estimate of HIV prevalence. Characteristics of the seeds—the starting point for RDS recruitment—also present a potential source of bias. In this regard it is encouraging that seeds tended to be older than the final sample (27 of 36 seeds were older than 24 years of age) and had higher educational attainment (35 of 36 seeds had completed secondary education while 22.1% of the final sample had completed at most primary education). However, assessment of bias is limited as the underlying demographic distribution of sexually active MSM in the cities surveyed is unknown.

## Conclusion

Our findings are a call to action for HIV prevention in Colombia, and signal, together with similar findings from Peru and Ecuador [37,47], an ongoing and likely undiminished HIV epidemic among MSM throughout the Andean region. Elevated HIV prevalence, significant sexual and drug risk behaviors, limited coverage of prevention and testing suggest that local adaptation and rapid scale-up of available proven risk reduction interventions [48] is needed. Interventions should aim to guarantee universal access to HIV testing, condoms and safe sex messaging, and should effectively engage MSM who frequent both physical venues and Internet meet-up services.

## Supporting Information

S1 File Table A and Table B. (DOCX)
