# Supplementary material for: High HIV Burden in Men Who Have Sex with Men across Colombia’s Largest Cities: Findings from an Integrated Biological and Behavioral Surveillance Study
Source: PLoS One. 2015 Aug 7;10(8):e0131040. doi: 10.1371/journal.pone.0131040 (PMC4529092; doi:10.1371/journal.pone.0131040)
Supplement: S1 File — (DOCX) [file pone.0131040.s001.docx]

**Table A. Male population and sample size of men who have sex with men by study site**

| Study site | Male populationaged ≥ 18 yearsin 2010 (thousands) ^[^[^49^](#_ENREF_49)^49]^ | Months ofdata collection(2010) | Plannedsample size | Sampleattained^§^ |
| --- | --- | --- | --- | --- |
| Andean Highlands |  |  |  |  |
| Bogota | 2420 | Jun-Nov | 504 | 488 |
| Medellin | 807 | May-Sep | 350 | 349 |
| Cucuta | 189 | May-Aug | 350 | 350 |
|  |  |  |  |  |
| Pacific Coast |  |  |  |  |
| Cali | 732 | May-Sep | 350 | 333 |
|  |  |  |  |  |
| Atlantic Coast |  |  |  |  |
| Barranquilla | 383 | May-Jun | 350 | 350 |
| Cartagena | 294 | May-Aug | 350 | 350 |
|  |  |  |  |  |
| Coffee Triangle |  |  |  |  |
| Pereira | 150 | May-Jun | 350 | 347 |
|  |  |  |  |  |
| All study sites | 4974 | May-Nov | 2604 | 2567 |

Notes: ^§^ Excludes seeds and participants not meeting study eligibility criteria.

**Table B. Characteristics of seed participants in the 7 study cities**

|  | **Barranquilla**  **(N=3)** | **Bogota**  **(N=9)** | **Cali**  **(N=5)** | **Cartagena**  **(N=4)** | **Cucuta**  **(N=6)** | **Medellin**  **(N=5)** | **Pereira**  **(N=4)** | **All study sites**  **(N=36)** |
| --- | --- | --- | --- | --- | --- | --- | --- | --- |
| **Age** |  |  |  |  |  |  |  |  |
| 18-24 | 0 | 1 | 2 | 1 | 1 | 1 | 3 | 9 |
| 25-34 | 1 | 3 | 1 | 1 | 3 | 1 | 1 | 11 |
| ≥ 35 | 2 | 5 | 2 | 2 | 2 | 3 | 0 | 16 |
| **Highest education attained** |  |  |  |  |  |  |  |  |
| Primary or below | 0 | 0 | 0 | 0 | 0 | 1 | 0 | 1 |
| Secondary | 0 | 3 | 4 | 1 | 5 | 2 | 3 | 18 |
| Vocational, university or above | 3 | 6 | 1 | 3 | 1 | 2 | 1 | 17 |
| **HIV infection**^§^ |  |  |  |  |  |  |  |  |
| Negative | 2 | 7 | 5 | 3 | 5 | 3 | 4 | 29 |
| Positive | 1 | 2 | 0 | 1 | 1 | 1 | 0 | 6 |

Notes: ^§^ HIV infection was indeterminate for one Medellin participant.
